# Probiotic Whey-Based Beverages from Cow, Sheep and Goat Milk: Antioxidant Activity, Culture Viability, Amino Acid Contents

**DOI:** 10.3390/foods12030610

**Published:** 2023-02-01

**Authors:** Nayil Dinkçi, Vildan Akdeniz, Ayşe Sibel Akalın

**Affiliations:** Department of Dairy Technology, Faculty of Agriculture, Ege University, Izmir 35100, Turkey

**Keywords:** whey valorization, goat WPC, sheep WPC, functional probiotic beverage, antioxidant activity, free amino acids

## Abstract

Recently, the demand for goat and sheep cheese has increased mainly because of its nutritional and health benefits. As a result, an enormous amount of whey from various animal species is produced as a waste/by-product. The production of functional probiotic fermented beverages from different types of whey protein concentrates (WPC) could be a good way to valorize whey. Meanwhile, reduced environmental pollution and economic sustainability will be provided. In this study, probiotic beverages enriched with 1% kiwi powder were produced from goat, sheep, and cow WPC (15%). Moreover, *Streptococcus salivarius* subsp. *thermophilus* and the probiotic bacteria *Lactobacillus acidophilus* and *Bifidobacterium animalis* subsp. *lactis* were used for fermentation. The results showed that WPC significantly increased the protein content and acidity of beverages (*p* < 0.05). Production with WPC also improved the viability of probiotic bacteria and *S. thermophilus*, total phenolic compound (TPC), and antioxidant activity of beverages. The highest viability of probiotic bacteria (9.67 log CFU/mL for Bb-12 and, 9.35 log CFU/mL for *L. acidophilus*) was found in beverages produced from goat WPC. In addition, WPC increased the free amino acid content of beverages, and the highest essential amino acids and branched-chain amino acids were found in beverages produced from goat WPC as 146.19 mg/100 g and 70.31 mg/100 g, respectively (*p* < 0.05). Consequently, while production with goat, cow, and sheep WPC improved quality compared to the control, beverages produced from goat WPC excelled. The production of a functional probiotic beverage with goat WPC is promising for dairy technology.

## 1. Introduction

One of the most important problems in the food industry is a large amount of by-products/waste-products generated during production [1]. The main by-product and as well liquid waste of the dairy industry, cheese whey, is released during cheese production and constitutes approximately 85–90% of the volume of milk used. It also contains about 55% of milk nutrients and 20% of milk proteins [2,3]. The worldwide production of cheese whey is estimated at nearly 200 million tons [4]. Despite this, in developed countries, whey is valorized into value-added products such as ricotta cheese, whey protein concentrates, whey protein isolates, and whey-based fermented beverages; especially in developing countries, most of the whey is still discharged into water sources without any treatment. In addition to the economic losses, it causes a serious environmental problem for the dairy industry for decades because of its high chemical and biological oxygen demand. [5]. However, whey proteins have high protein efficiency, net protein utilization, and biological value compared to all other protein sources [6]. In addition, whey proteins have health-promoting properties such as antimicrobial, anti-cancerogenic, immune-stimulating, and antioxidant activities; reduction in blood pressure, the risk of cardiovascular disease and osteoporosis; and satiety regulation. Due to these properties, it is a valuable by-product [7,8,9].

In world milk production, although cow’s milk (751.7 Mt) is in the leading position, sheep milk (10.4 Mt) and goat milk (15.3 Mt) has also increased. [10,11]. These sheep’s and goat’s milk are mainly utilized for the manufacture of excellent artisanal cheeses [12] which are gained popularity among conscious consumers depending on their special flavor, texture, and also nutritional and health effects. Thus, the amount of cheese wheys of sheep and goat milk produced worldwide has also increased [13]. The animal species is one of the most important factors affecting the whey composition [14]. Therefore, goat, sheep, and cow whey have differences, especially in amino acid composition. Goat cheese whey contains higher concentrations of oligosaccharides such as sialic acid, which is considered that promotes brain development in infants, compared to cow and sheep whey [15]. It is rich in nucleotides (nonprotein nitrogen compounds) and essential amino acids [16]. Milk from small ruminants is potentially less allergenic and whey from goats and sheep can also be used in the manufacture of products for people (including children) with cow’s milk intolerance [16]. For these reasons, the valorization of sheep and goat whey into value-added products is becoming increasingly important [17].

Ready-to-drink beverages are among the preferred functional foods which meet consumer demand for nutrients and bioactive molecules such as vitamins, minerals, antioxidants, fatty acids, and fiber [18]. In this context, the popularity of value-added beverages, especially high-protein beverages, is increasing in the world [19], and whey-based beverages are a perfect way to valorize whey into high-value-added products. However, whey is highly perishable because of its high water content [14], and there are difficulties in transporting and storing whey because of its high volume [20]. Therefore, as in the studies by Pescuma et al. [3] and Rocha et al. [21], it is a good option to use whey in beverage production as whey protein concentrate (WPC), which is considered a valuable food ingredient for the functional food market. In the meantime, it also contributes to the production of high-protein and lactose-free beverages. Whey proteins have superior biological value compared to other proteins, and also contain all essential amino acids, highly branched-chain amino acids (BCAAs), and highly sulfur-containing amino acids, which support antioxidant activities. These amino acids also induce the synthesis of the intracellular antioxidant, glutathione [2,22,23]. Recent research results indicate that whey proteins deserve more attention as a valuable raw material for the production of functional foods due to these properties [7,9,24]. Antioxidants are known to provide health advantages to consumers by combating oxidative stress and reducing the risk of diseases such as cardiovascular and inflammatory diseases [24].

Whey-based beverages are also an effective alternative for probiotic bacteria intake which offers tremendous health potential when consumed in adequate amounts [18,25]. Whey proteins enhance the growth of probiotic bacteria and protect them from oxidative stress due to the antioxidant effects of whey proteins [26]. Furthermore, the functionality of whey beverages can be increased by enrichment with various powders and flavors from different functional fruits and vegetables [27]. Kiwi is one of these fruits as it contains vital substances that are important for health. It contains vitamins C, E, sugar, phosphorus, potassium, magnesium, copper, calcium, and high levels of bioactive compounds such as ascorbic acid, total phenols, anthocyanins, chlorophylls, carotenoids, tannins, flavanols, and flavonoids. Kiwi provides health benefits due to its nutritional content and rich antioxidant activity [28,29]. Therefore, in this study whey-based beverages were enriched with kiwi powder.

Although there are limited studies on the production of whey-based probiotic beverages, no studies were found that compared the effect of different origins of WPC on product quality criteria, and also the FAA and EAA content of the beverages. In this context, the aim of this study was to produce probiotic fermented whey-based beverages enriched with kiwifruit powder from cow, sheep, and goat WPC, and to evaluate the effects of different types of WPC on probiotic bacteria viability, antioxidant activity, total phenolic compound (TPC), the amino acid content of the probiotic beverages during storage. 

## 2. Materials and Methods

### 2.1. Materials

The commercial Nutrish ABY Permium Probiotic Culture containing *Streptococcus thermophilus*, *Lactobacillus acidophius* LA-5, and *Bifidobacterium animalis* subsp. *Lactis* Bb-12 (Chr. Hansen, Denmark) was used in the study. The cow, sheep, and goat WPC60 including >60% protein and <5% moisture were provided by Alimenta Srl (Macomer, Italy). Kiwi powder with >95% total solids was obtained from Fx Food (Antalya, Türkiye). Liquid kiwi flavor was supplied by Aromsa (Kocaeli, Türkiye). Sugar was purchased from a local market. Curcumin with bright yellow and copper complexes of chlorophyll with a natural green color was used as a coloring agent (Gemma, Kocaeli, Türkiye).

### 2.2. Manufacture of Probiotic Whey Beverages Enriched with Kiwi Powder

Probiotic-fermented whey beverages were manufactured from a cow (group C), sheep (group S), and goat WPC (group G), separately. WPC amounts in the beverages were determined as 15% after pre-trials, 5% sucrose, 1% kiwi powder, coloring agent (0.036% Curcumin + 0.012% Copper Complexes of Chlorophyll), 0.08% liquid kiwi flavor were added to the groups. Additionally, as a control, group W was produced with the same ingredients without WPC. After water addition, pasteurization was carried out at 70 °C for 15 min followed by cooling to inoculation temperature (42 °C). Nutrish ABY Permium Culture (0.7%) was inoculated into the pasteurized mixture. Then incubation was carried at 42 °C until pH 4.75 was reached. The beverages were then cooled and stored at 4 °C over 28 days for microbiological, antioxidant, and free amino acid analyses. Analyses were carried out on d 1, d 14, and d 28 of storage except for acidity and microbiological analyses which were carried out weekly. The experiments were replicated two times.

### 2.3. Physicochemical Analysis

The protein content was determined according to the method described in the Association of Official Analytical Chemists methods [30]. Titratable acidity was expressed as g of lactic acid/100 g. Since the color change in beverages enriched with kiwi powder cannot be distinguished visually, the titration with 0.1 N sodium hydroxide after adding 1 mL of 1% phenolphthalein was carried out in the presence of a pH meter until reaching the endpoint, pH = 8.1 [30]. 

### 2.4. Microbiological Analysis

The bacterial enumerations in the probiotic whey beverages were performed using the pour plate technique. *Streptococcus salivarius* subsp. *Thermophilus* (*S. thermophilus*) was enumerated using M17 Agar (Merck, Darmstadt, Germany) and *Lactobacillus acidophilus* was enumerated using MRS-Sorbitol Agar (Oxoid Ltd., Hampshire, UK) according to Dave and Shah [31]. TOS-MUP Agar (Merck, Darmstadt, Germany) was used for *Bifidobacterium animalis* subsp. *Lactis* (Bb-12) enumeration according to Raeisi et al. [32]. Anaerobic jars (Aaerocult A, Merck, Darmstadt, Germany) were used for the incubation of bacteria, except *S. thermophilus* which was incubated under aerobic conditions. The incubation temperature for *S. thermophilus, L. acidophilus*, and Bb-12 was 37 °C for 48 h, 72 h, and 72 h, respectively. Cell counts were expressed as log cfug^−1^.

### 2.5. Total Phenolic Compound (TPC)

The total phenolic compound (TPC) content in beverages was determined following the Folin–Ciocalteau’s phenol reagent colorimetric method according to Kocazorbaz et al. [33]. Crude extracts of each sample were dissolved in methanol, and gallic acid was used as a standard. The results were expressed as mass (g) of Gallic Acid Equivalents (GAE) per gram of mass of the sample (g GAE/g).

### 2.6. Antioxidant Activity

In the foods, different methods such as 2,2-diphenyl-1-picrylhydrazyl radical-scavenging activity (DPPH), Cupric ion reducing antioxidant capacity (CUPRAC), ABTS (2,2-azinobis (3-etilbenzothiazollin-6-sulfonik asit)), and FRAP (ferric reducing antioxidant power) can be used to determine antioxidant activity. Each method had advantages and disadvantages over the other methods. While the antioxidant activity of hydrophilic substances can be measured using the DPPH method, the antioxidant activity of hydrophilic substances as well as hydrophobic substances can be measured with the CUPRAC method. Moreover, the CUPRAC radical is more stable than the chromogenic radical reactive DPPH [34]. Therefore, in this study, in addition to the DPPH method, which is commonly used for antioxidant activities, the CUPRAC method was used to determine the antioxidant activities of probiotic whey beverages.

#### 2.6.1. DPPH Radical Scavenging Activity

DPPH is a widely used method for the assessment of the antioxidant capacities of natural products. It is a spectrophotometric technique based on the quenching of stable colored radicals and demonstrates the radical scavenging ability of antioxidants, even in complex biological mixtures [35,36]. DPPH of whey beverages was determined according to Pavithra and Vadivukkarasi [37]. Ascorbic acid was used as a standard and the results were expressed as millimolar (mM) of Ascorbic Acid Equivalents (AAE) per gram of mass of the sample (mM AAE/g).

#### 2.6.2. Cupric Ion Reducing Antioxidant Capacity (CUPRAC)

CUPRAC assays of probiotic beverages were determined according to Apak et al. [38]. In the assay, bis(neocuproine) copper(II) chelate was used as a chromogenic redox reagent. Cu(I)-chelate was formed as a result of the reaction of bis(neocuproine)copper(II) chloride (Cu(II)-Nc) with n-electron-reducing antioxidants. The color resulting from the Cu(I)-Nc chelation was measured at 450 nm. Ascorbic acid was used as a standard and the calculations were expressed as millimolar (mM) of Ascorbic Acid Equivalents (mM AAE/g).

### 2.7. Free Amino Acids (FAAs) Analysis

The free amino acids (FAAs) analysis was performed on an HPLC Agilent 1260 Infinity II series chromatograph (Agilent Technologies, Morge, Switzerland). Separations were performed on the Agilent Eclipse AAA column. Solvent A was 40 mM Borate Buffer (pH adjusted to 7.8), and Solvent B was can:MeOH:water (45:45:10, *v*/*v*/*v*). The flow rate was 2 mL/min, the column temperature was 40 °C and the wavelength for the FLD detector was 338 nm. The method has been applied according to [39]. One milliliter of Sulfosalicylic Acid Solution was added to 1 g of homogenized sample and it was completed to 100 mL with water. After mixing, it was centrifuged at 4000 rpm for 5 min and passed through an O.45 µm nylon filter. One milliliter was transferred to a vial and 20 µL was injected into the HPLC system. The results were expressed as mg per 100 g of the sample (mg/100 g).

### 2.8. Statistical Analysis

In the study, two experimental replicates were carried out. The obtained data were processed by the one-way analysis of variance (ANOVA) in IBM SPSSv.25 (IBM SPSS, Armonk, NY, USA), and the mean differences were analyzed using Duncan’s multiple range test at the pre-set *p* < 0.05 level.

## 3. Results

### 3.1. Physicochemical Properties (Protein and Acidity)

**Figure 1:**
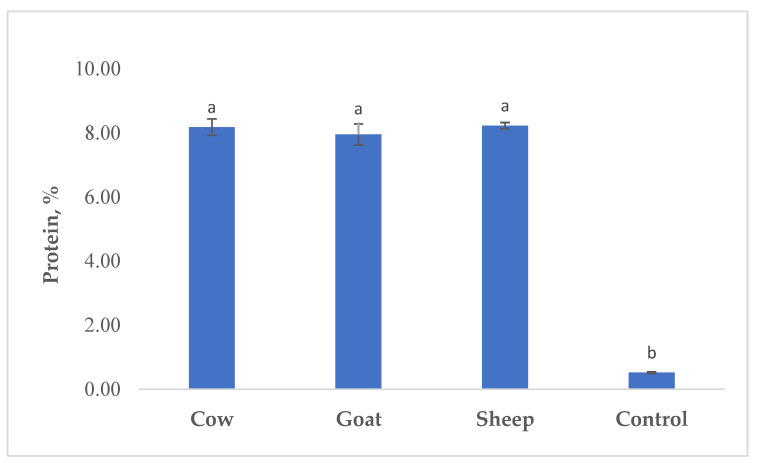
Protein content (%) in probiotic fermented whey beverages enriched with kiwi powder. ^a,b^ Means ± standard deviations with different superscript uppercase letters are significantly different (*p* < 0.05).

Figure 1 shows the protein content of beverages enriched with kiwi powder. As expected, the beverages produced with WPC had significantly higher protein content than control beverages (*p* < 0.05), and the type of WPC did not significantly affect protein values.

The titratable acidity values of beverages during storage are presented in Table 1. Variations were observed for either type of WPC and the storage time. The acidity values of beverages varied from 0.08 to 0.88 g of lactic acid/100 g. As seen in Table 1, great differences were obtained between the values of control and WPC-containing beverages. The control beverage had the lowest acidity values.

### 3.2. Microbiological Properties: Lactic Acid Bacteria and Probiotic Bacteria Viability

The microbiological attributes of beverages during the storage period are presented in Table 1. Bacteria viability was significantly influenced by both the type of WPC and the storage time (*p* < 0.05).

At the beginning of storage, Streptococcus salivarius subsp. Thermophilus counts varied between 7.08 and 9.39 log CFU/mL, while they were enumerated between 6.43 and 8.57 log CFU/mL at the end of storage. During the whole storage period, Bifidobacterium animalis subsp. lactis (Bb-12) counts ranged from 7.25 to 9.67 log CFU/mL and Lactobacillus acidophilus counts ranged from 5.98 to 9.35 log CFU/mL (Table 1). The highest numbers of S. thermophilus, Bb-12, and L. acidophilus were found in beverages produced from goat WPC on d 14, d 7, and in beverages produced from goat and sheep WPC on d 14 of storage, respectively. However, the lowest numbers for all bacteria were found in control samples produced without WPC throughout the storage.

### 3.3. Total Phenolic Compound and Antioxidant Activity

**Figure 2:**
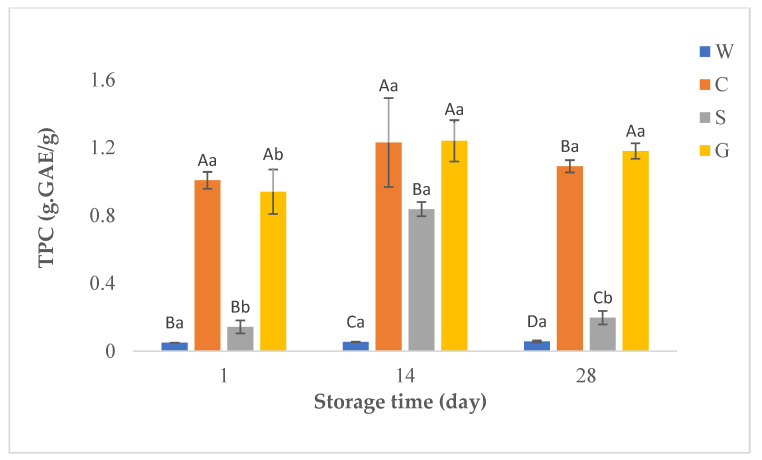
TPC (g.GAE/g) in probiotic fermented whey beverages enriched with kiwi powder. ^a,b^ Means ± standard deviations in the same beverage group with different superscript lowercase letters are significantly different (*p* < 0.05); ^A–D^ Means ± standard deviations in the same storage day with different superscript uppercase letters are significantly different (*p* < 0.05). C = probiotic fermented beverage produced from cow WPC and enriched with kiwi powder, G = probiotic fermented beverage produced from goat WPC and enriched with kiwi powder, S = probiotic fermented beverage produced from sheep WPC and enriched with kiwi powder, W = probiotic fermented beverage produced without WPC and enriched with kiwi powder.

**Figure 3:**
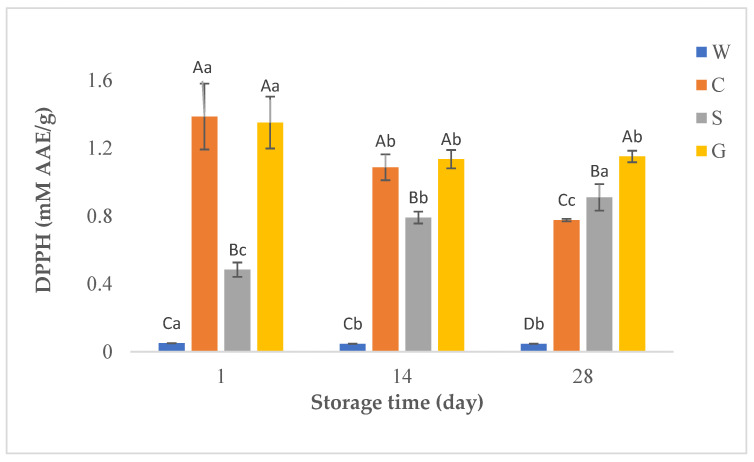
DPPH (mM AAE/g) in probiotic fermented whey beverages enriched with kiwi powder. ^a–c^ Means ± standard deviations in the same beverage group with different superscript lowercase letters are significantly different (*p* < 0.05); ^A–D^ Means ± standard deviations in the same storage day with different superscript uppercase letters are significantly different (*p* < 0.05). C = probiotic fermented beverage produced from cow WPC and enriched with kiwi powder, G = probiotic fermented beverage produced from goat WPC and enriched with kiwi powder, S = probiotic fermented beverage produced from sheep WPC and enriched with kiwi powder, W = probiotic fermented beverage produced without WPC and enriched with kiwi powder.

**Figure 4:**
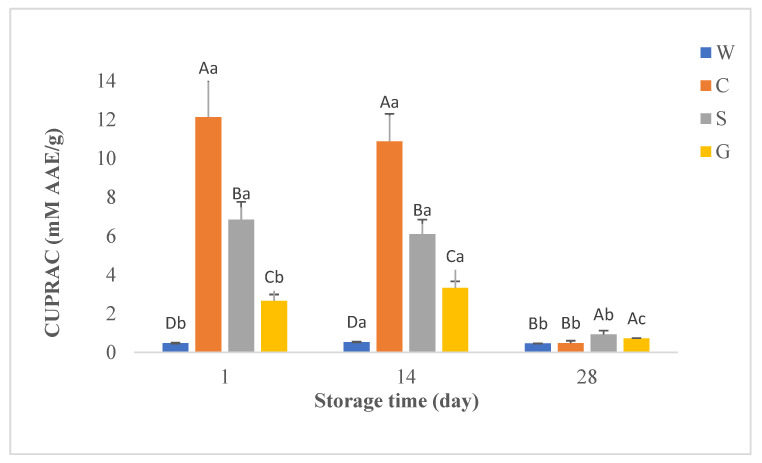
CUPRAC (mM AAE/g) in probiotic fermented whey beverages enriched with kiwi powder. ^a–c^ Means ± standard deviations in the same beverage group with different superscript lowercase letters are significantly different (*p* < 0.05); ^A–D^ Means ± standard deviations in the same storage day with different superscript uppercase letters are significantly different (*p* < 0.05). C = probiotic fermented beverage produced from cow WPC and enriched with kiwi powder, G = probiotic fermented beverage produced from goat WPC and enriched with kiwi powder, S = probiotic fermented beverage produced from sheep WPC and enriched with kiwi powder, W = probiotic fermented beverage produced without WPC and enriched with kiwi powder.

In this study, antioxidant activities of whey beverages were determined using DPPH, and CUPRAC methods. Figure 2, Figure 3 and Figure 4 show the results of TPC and antioxidant activity assays of probiotic whey beverages enriched with kiwi powder.

#### 3.3.1. Total Phenolic Compound (TPC)

The principle of the Folin–Ciocalteu test is the reduction of the Folin–Ciocalteu reagent in an alkaline state by phenolic compounds. The Folin–Ciocalteu reagent has a phosphomolybdic/phosphotungstic acid complex which is reduced to the blue-colored complexes by electron transfer from phenolic compounds at 765 nm [40]. Figure 2 shows the TPC results of probiotic whey beverages enriched with kiwi powder.

WPC from different milk types has affected the TPC of beverages. However, prolonged storage did not cause any significant differences (*p* > 0.05) in the TPC of control beverages and beverages produced from cow WPC, while fluctuations were observed for beverages produced from cow and goat WPC. The highest TPC value was found in the beverages produced from goat WPC on d 14 of storage. In general, the beverages produced with goat WPC had higher TPC values throughout storage. However, the differences for beverages from goat and cow WPC were significant only on d 28 (*p* < 0.05). As expected, control samples had the lowest values during the whole storage, and the differences were significant on d 14 and d 28 between control samples and beverages produced from sheep WPC (*p* < 0.05). 

#### 3.3.2. 2,2-Diphenyl-1-Picrylhydrazyl Radical-Scavenging Capacity (DPPH)

DPPH, a stable nitrogen-based free radical, has a violet color. However, it is reduced to a yellow-colored diphenylpicrylhydrazine radical, which can be measured colorimetrically, by accepting an electron from the antioxidant compound. Thus, antioxidants that are able to carry out this reaction are considered radical scavengers [41]. Figure 3 shows the DPPH results of probiotic whey beverages enriched with kiwi powder. The use of whey powder concentrates from different types of milk showed significant differences (*p* < 0.05) in DPPH results both between samples containing WPC and also compared to the control sample. Consistent with the TPC results, the highest DPPH values were detected in the beverages obtained from goat and cow WPC, while the lowest values were found in the control sample without WPC throughout the storage.

#### 3.3.3. Cupric Ion Reducing Antioxidant Capacity assay (CUPRAC)

The antioxidant activity values of probiotic whey beverages enriched with kiwi powder obtained by the CUPRAC method are given in Figure 4. Regarding the CUPRAC test, the antioxidant activity of the control samples was the lowest throughout the storage in parallel with DPPH values. The results showed that production with WPC increased the CUPRAC values and significant differences were observed between these beverages. The beverages produced from cow WPC had the highest values on d 1 and d 14, while the beverages produced from sheep and goat WPC on d 28 had the highest values.

Pearson correlation between TPC and antioxidant capacity methods is presented in Table 2. There was a positive high correlation between the TPC and DPPH method (*p* < 0.01), while a moderate positive correlation was found between the DPPH and CUPRAC methods (*p* < 0.05). However, there wasn’t a significant correlation between the TPC and CUPRAC method.

### 3.4. Free Amino Acids (FAAs)

Amino acids have vital importance for humans [42]. The profile and amount of amino acids in probiotic whey beverages enriched with kiwi powder are given in Table 3. The results indicate that the WPC type had a pronounced effect on the contents of amino acids. The total FAAs of the probiotic beverages enriched with kiwi powder ranged from 518.02 to 799.29 mg/100 g. The highest total FAAs were observed in the beverage produced from sheep whey at the beginning of storage, while the beverages produced from goat whey had the highest total FAAs on d 14 and d 28. The lowest total FAAs belonged to beverages produced without WPC throughout the storage. Furthermore, at the beginning of storage, essential amino acids (EAAs) were detected at the highest concentration in beverages from goat and cow WPC, while the lowest concentration was found in beverages without WPC. On the other storage days, probiotic beverages produced from goat WPC had the highest EAAs. This was followed by beverages from cow and sheep WPC, respectively. The lowest values were observed in beverages without WPC. The BCAAs content of beverages was determined from high to low concentrations in the samples produced from goat, cow, and sheep WPC and without WPC, respectively.

Pearson correlations between FAAs, EAAs, TPC, and antioxidant capacity methods are presented in Table 4. There were significant positive high correlations between the EAAs and TPC (r = 0.821) and DPPH method (r  =  0.846) (*p* < 0.01), while a significant moderate positive correlation was found between the FAAs and TPC (r = 0.416 *) and DPPH method (r = 0.572 **). In addition, a significant moderate positive correlation (r = 0.615) was found between the EAAs and FAAs (*p* < 0.01). However, there wasn’t a significant correlation between the CUPRAC method and the EAAs and FAAs. 

## 4. Discussion

Whey protein is one of the high-quality food proteins with a full spectrum of amino acids, including EAAs and BCAAs, which are important for tissue growth and repair [43]. As expected, the control beverage without WPC had very low protein content compared to the beverages produced from different types of WPC containing 60% protein. They had a protein content of about 8.12%, while it was only 0.52% in the control. Since WPC60 (cow, goat, sheep) was used in equal proportions in the product formulation, there was no significant difference in the protein content of the beverages produced from different WPC types.

The production of beverages enriched with kiwi powder using WPC affected the acidity of the beverages. The beverages produced from different types of WPC had acidity values ranging from 0.76 to 0.85 g lactic acid/100 g, while the control sample without WPC had 0.08 and 0.09 g lactic acid/100 g. The lowest acidity values were obtained in the control samples throughout storage. At the beginning of storage, the highest lactic acid values were determined in the beverages produced from sheep WPC, while at the end of storage, the beverages produced from goat WPC had the highest values. Nine amino acids (i.e., phenylalanine, valine, threonine, tryptophan, methionine, leucine, isoleucine, lysine, and histidine) which cannot be synthesized by humans are defined as essential amino acids and must be absorbed from foods. Whey proteins are unique high-quality proteins as they contain all essential amino acids, and branched-chain amino acids (i.e., leucine, isoleucine, and valine). In addition, their concentrations in whey are higher than those of various plant protein sources [6,42]. Rocha et al. [21] reported that proteins and amino acids contribute to increasing acidity in foods, and it is well-known that whey proteins are rich in amino acids. The results of this study support this statement in relation to total FAAs. Additionally, the conversion of lactose and proteins in whey-based beverages to lactic acid and amino acids leads to an increase in acidity [44,45]. Similarly, higher acidity had been found in whey-based beverages compared to the control by Thakkar et al. [44] and Naik et al. [46]. Moreover, storage time affected the acidity values of the beverages, and the fluctuations observed during the storage period. This could be due to the growth cycle of lactic acid and probiotic bacteria in the beverages.

Regarding microbiological aspects, although some fluctuations in bacteria counts were detected for all samples during storage, at the end of storage it was detected that the viability of *S. thermophilus*, Bb-12, and *L. acidophilus* generally decreased compared to the beginning of storage, but the difference was not significant for the individual beverages. In terms of *S. thermophilus* viability, at the beginning of storage, in parallel to the acidity values, the beverages produced from sheep WPC had the highest value, while there was no significant difference between beverages produced from different types of WPC on d 21 and d 28 of storage (*p* > 0.05). The highest *S. thermophilus* counts were found in beverages produced from goat WPC on d 14 of storage. Control samples had lower values than beverages including WPC throughout the storage. During the whole storage period, viable Bb-12 counts were higher in all WPC-based beverages than in the control sample. The highest values were enumerated in the beverages produced from sheep WPC on d 1, while beverages produced from goat WPC had the highest values on the other storage days. However, the differences between beverages produced from goat WPC and beverages produced from cow WPC/sheep WPC were significant only on d 7. Similar to Bb-12 probiotic bacteria counts, *L. acidophilus* probiotic bacteria counts were higher in the beverages produced from WPC than in the control samples throughout the storage (*p* < 0.05). The differences between beverages produced from different types of WPC were significant only on d 14 and d 21. The highest values were determined in beverages produced from both sheep and goat WPC and beverages produced from goat WPC on d 14 and d 21, respectively. In addition, the control beverages could not maintain *L. acidophilus* at an adequate level (>7 log CFU/mL) for the probiotic product from d 7 on storage. It is obviously observed that production using WPC improved the counts of *S. thermophilus*, Bb-12, and *L. acidophilus* in probiotic whey beverages enriched with kiwi powder during storage. The higher viability of probiotic bacteria and *S. thermophilus* in beverages produced with WPC may be associated with the consumption of protein fractions present in WPC by bacteria both during the fermentation process and during storage [47]. Researchers have already stated that whey is a good medium for the growth of probiotic bacteria [26,48,49,50]. Whey proteins protect probiotic bacteria against oxidative stress since they include sulfur-containing amino acids. In addition, whey proteins stabilize pH depending on their buffering capacity, which leads to better survival for culture bacteria [48,51]. 

Consistent with the results of this study, Skryplonek and Jasińska [52] found that the counts of *L. acidophilus* and Bb-12 were above 8 log CFU/mL in the probiotic beverage developed from fresh acid cheese whey supplemented with sweet whey powder during the 21 days storage period. Similarly, Bulatović et al. [50] and Pereira et al. [53] found that the counts of probiotic bacteria in the whey-based beverage were above 7 log CFU/mL, which is the therapeutic minimum level during 21 days and 14 days of cold storage, respectively. Thakkar et al. [44] reported that orange-whey juice had higher and adequate counts of probiotic bacteria, while the control juice without whey did not have adequate numbers of probiotic bacteria.

The characteristics and composition of whey protein closely depend on the source of milk (cow, sheep, goat, etc.) [6]. The type of WPC has a remarkable impact on TPC, antioxidant activity, and total FAAs and EAAs content in beverages. In this study, WPC from different types of milk affected the TPC and antioxidant activity of the beverages. Probiotic beverages produced with goat and cow WPC had the highest TPC and DPPH values at the first and 14th days, while the goat WPC-based product had the highest values at the end of storage (*p <* 0.05). Beverages produced without WPC had the lowest TPC and DPPH values throughout storage. In addition, a significant positive high correlation (r = 0.820) was found between TPC and DPPH values. According to the CUPRAC assay results, the antioxidant activity of the control samples was the lowest during storage, which is consistent with the DPPH values. The phenolic content in beverages is closely related to their antioxidant function, and they are important in determining antioxidant activity since they can capture reactive oxygen and nitrogen species by donating an electron or hydrogen atom in the molecule. Moreover, they could interact with proteins generating soluble or insoluble complexes which could alter the antioxidant capacity of the components [54,55]. Thus, it is an expected result that the control samples without WPC had the lowest TPC, DPPH, and CUPRAC values. On the other hand, beverages with high CUPRAC values had some discrepancies compared to TPC and DPPH values, and no significant correlation was found between TPC and CUPRAC values. The reason for this might be due to the different method mechanisms. Therefore, in this study, it was concluded that it is more appropriate to use the DPPH method to determine the antioxidant activity of beverages. The storage period also affected the TPC values and antioxidant capacities of beverages produced from cow and goat WPC. The observed fluctuations may result from the decomposition of polymeric phenols in the presence of lactic acid bacteria during storage.

The interaction between whey proteins and phenolic compounds also affects antioxidant capacity, either positively or negatively [56,57,58]. These contradictory effects are attributed to the differences in antioxidant assays, the type of proteins and phenolic compounds, and the ratio between proteins and phenolic compounds [54]. Campos et al. [14] reported that the antioxidant activity of the samples increased with the increase in WPC concentrations in the samples. It is considered that β-lactoglobulin, α-lactalbumin, bovine serum albumin, immunoglobulins, and lactoferrin in whey are the main factors responsible for the antioxidant activity [24]. Whey proteins in cow, sheep, and goat milk are 6.46, 10.76, and 6.14 g/L, respectively [9], and the protein concentration of protein fractions including α-lactalbumin, β-lactoglobulin, lactoferrin, lysozyme, immunoglobulins, and serum albumin varies significantly across these milk types [59]. Since WPC from cow, goat, and sheep had different compositions, there were differences in the results of TPC, DPPH, and CUPRAC of the beverages from WPC of the different milk types.

Srivastava et al. [60] found that goat yogurt enriched with herbs had higher antioxidant activity than cow yogurt enriched with the same herbs, using the DPPH method. Similarly, Balakrishnan and Agrawal [61] determined higher antioxidant activity in probiotic fermented products produced from goat milk, compared to probiotic fermented products from cow and camel milk. Simos et al. [62] reported that the antioxidant activity of milk from Prisca goats was higher than the antioxidant activity of cow milk. It can be seen that the results of this study are compatible with the results of the aforementioned studies. In the same way, Öztürk and Akın [63] found high antioxidant activity in Tulum cheese produced from goat milk and it was attributed to the presence of highly hydrophilic peptides in goat milk. Mushtaq et al. [64] reported that the antioxidant activity determined by the DPPH method was higher in cheese produced from goat milk than in cheese produced from buffalo milk. The high antioxidant capacity of goat milk was attributed to the particular combination or higher bioavailability of the compounds, as well as to its rich antioxidant composition. In addition, Nishino et al. [65] stated that the radical scavenging activity increased due to the protein peptides present in the fermented milk products. Thus, the high antioxidant activity of goat whey is considered to be due to its numerous numbers of small peptides. Accordingly, Marcillo et al. [66] reported that the antioxidant activity values of goat whey beverages were significantly higher in the samples containing a higher amount of goat whey depending on the possible presence of small peptides in goat whey. In addition, the higher antioxidant activity results in beverages produced from goat and cow WPC may be due to the higher presence of cysteine and methionine amino acids in goat and cow WPC. It is a well-known fact that thiol (R-SH) Cys reacts readily with many antioxidants [14]. 

Whey protein includes sulfur-containing amino acids such as cysteine and methionine in abundance and in balance. These amino acids exhibit antioxidant activity by acting directly as reducing agents or promoting glutathione synthesis which is an important intracellular antioxidant [2,23]. In addition, amino acids such as tyrosine, tryptophan, and lysine have antioxidant properties since their sulfhydryl–SH groups interact directly with radicals [67]. In this context, the results of amino acid contents in this study were in parallel with the results of TPC and antioxidant activity. While methionine as an antioxidant component was detected in high concentration in beverages produced from cow WPC (6.64–17.47 mg/100 g) and goat WPC (6.01–10.86 g/100 g), it could not be detected in beverages produced without WPC and sheep WPC. Cysteine, which enhances the antioxidant activity, was also in higher concentration in beverages produced from goat WPC and this was followed by beverages produced from cow WPC. Whereas beverages produced from sheep WPC or without WPC had lower concentrations. These results support TPC and antioxidant activity results. Moreover, positive high correlations were found between EAAs and TPC and antioxidant activity determined by the DPPH method.

As expected, beverages produced without WPC had the lowest FAAs and EAAs contents throughout the storage. Higher contents of FAAs and EAAs in beverages produced with WPC could be associated with the protein-derived present in whey, and also the release of new peptides by probiotic and lactic cultures [47]. The results of the study showed that beverages produced from goat WPC had the highest EAAs content. In addition, the EAAs content of beverages produced from cow WPC was close to these values, especially on d 1 of the storage. Total FAAs contents of beverages produced from goat WPC were also higher on d 14 and d 28 of the storage, while beverages without WPC had the lowest values throughout storage. 

Although there is limited research on the amino acid composition of WPC from different types of milk, Ayunta et al. [68] found that methionine in caprine WPC was higher than bovine WPC. Garay et al. [69] reported that whey protein fortified drink based on goat whey contained all EAAs and BCAAs in high content. Landi et al. [70] reported that free amino acid profiles obtained from cow, sheep, and goat raw milk are characteristic. Çayır and Güzeler [71] indicated that the total FAAs values of the product increased with an increase in the goat milk ratio in the product. Macedo et al. [72] informed that goat cheese whey had higher free amino acids compared to bovine or sheep cheese whey. 

Branched-chain amino acids, e.g., leucine, isoleucine, and valine, are important for human nutrition as they participate in protein and glucose homeostasis, enhance growth hormone circulation, decrease lactate levels, stimulate insulin release, and control blood sugar levels [68,73]. In the study, similar to the contents of FAAs and EAAs, beverages produced without WPC had the lowest content of BCAAs, while goat WPC-based beverages had the highest contents throughout the storage. 

Furthermore, the storage period has significantly affected the content of total FAAs and EAAs in the beverages. LAB, which plays an important role in the degradation of peptides to produce free amino acids, is considered to be responsible for the fluctuations in these variations of contents [74].

## 5. Conclusions

The use of WPC and the origins of WPC used affected the quality of probiotic beverages enriched with kiwi powder. WPC provided higher protein content and acidity in the beverages than in control samples. WPC also improved the viability of *S. thermophilus* and probiotic bacteria throughout the storage. In addition, WPC contributed to the increase in FAA, EAA, and BCAA contents as well as TPC content and the antioxidant capacity of beverages. A positive high correlation between the TPC and DPPH methods was found, and it was concluded that the DPPH method is more appropriate for determining the antioxidant activity of beverages. Within WPC-based probiotic beverages, beverages from goat WPC indicated the highest viable counts of probiotic bacteria and antioxidant activity as well as EAA and BCAA contents. 

Thus, the findings demonstrated that different origins of WPC could be utilized for probiotic beverage production with good outcomes. Especially, goat WPC, which is less valorized in the dairy industry, has a high potential for use in a functional food market. Further studies could focus on the effect of different types of WPC, especially goat WPC, on other product quality criteria such as texture and sensory properties of beverages.

## Figures and Tables

**Table 1:** Lactic acid (%) and viable bacteria count (log CFU/mL) changes in probiotic fermented whey beverages enriched with kiwi powder during storage.

Item	Sample	Storage Days				
		1	7	14	21	28
Lactic acid (%)	C	0.80 ± 0.01 ^Ad^	0.85 ± 0.01 ^Ab^	0.88 ± 0.01 ^Aa^	0.88 ± 0.02 ^Aa^	0.83 ± 0.00 ^Ac^
	G	0.76 ± 0.03 ^Bc^	0.85 ± 0.01 ^ABa^	0.81 ± 0.01 ^Cb^	0.85 ± 0.01 ^Ba^	0.85 ± 0.01 ^Aa^
	S	0.82 ± 0.01 ^Aa^	0.84 ± 0.00 ^Ba^	0.83 ± 0.01 ^Ba^	0.77 ± 0.01 ^Cc^	0.80 ± 0.03 ^Bb^
	W	0.08 ± 0.00 ^Cb^	0.09 ± 0.00 ^Ca^	0.09 ± 0.00 ^Da^	0.08 ± 0.00 ^Db^	0.08 ± 0.00 ^Cb^
*Streptococcus thermophilus*	C	8.21 ± 0.23 ^Cc^	9.18 ± 0.26 ^ABa^	8.65 ± 0.18 ^Bb^	8.56 ± 0.14 ^Abc^	8.44 ± 0.04 ^Abc^
	G	8.66 ± 0.08 ^Bb^	9.81 ± 0.98 ^Aa^	10.03 ± 0.05 ^Aa^	8.42 ± 0.05 ^Ab^	8.57 ± 0.04 ^Ab^
	S	9.39 ± 0.16 ^Aa^	8.54 ± 0.08 ^Bb^	9.06 ± 0.37 ^Ba^	8.39 ± 0.08 ^Ab^	8.51 ± 0.18 ^Ab^
	W	7.08 ± 0.10 ^Dab^	7.00 ± 0.00 ^Cab^	7.48 ± 0.91 ^Cab^	7.73 ± 0.66 ^Ba^	6.43 ± 0.51 ^Bb^
*Bifidobacterium animalis* subsp. *lactis* (Bb-12)	C	8.59 ± 0.08 ^BCb^	8.90 ± 0.30 ^Ba^	8.56 ± 0.07 ^ABb^	8.62 ± 0.07 ^Ab^	8.53 ± 0.06 ^Ab^
	G	8.75 ± 0.05 ^Bb^	9.67 ± 0.32 ^Aa^	9.11 ± 0.98 ^Aab^	8.74 ± 0.08 ^Ab^	8.53 ± 0.05 ^Ab^
	S	9.63 ± 0.13 ^Aa^	8.49 ± 0.01 ^Bc^	9.05 ± 0.09 ^Ab^	8.47 ± 0.03 ^Bc^	8.44 ± 0.03 ^Ac^
	W	8.50 ± 0.03 ^Ca^	7.60 ± 0.13 ^Cc^	7.98 ± 0.02 ^Bb^	7.41 ± 0.10 ^Ccd^	7.25 ± 0.22 ^Bd^
*Lactobacillus acidophilus*	C	8.61 ± 0.02 ^ABa^	8.10 ± 0.35 ^Ab^	8.29 ± 0.07 ^Bab^	8.31 ± 0.04 ^Bab^	8.23 ± 0.12 ^Ab^
	G	9.15 ± 0.43 ^Aab^	8.17 ± 0.02 ^Ad^	9.35 ± 0.30 ^Aa^	8.75 ± 0.11 ^Abc^	8.57 ± 0.05 ^Acd^
	S	9.07 ± 0.44 ^Aa^	7.93 ± 0.63 ^Ab^	9.03 ± 0.18 ^Aa^	8.31 ± 0.01 ^Bb^	8.18 ± 0.06 ^Ab^
	W	8.24 ± 0.05 ^Ba^	6.16 ± 0.09 ^Bb^	5.98 ± 0.05 ^Cb^	6.00 ± 0.01 ^Cb^	6.30 ± 0.52 ^Bb^

^a–d^ Means ± standard deviations in the same row with different superscript lowercase letters are significantly different (*p* < 0.05); ^A–D^ Means ± standard deviations in the same column with different superscript uppercase letters are significantly different (*p* < 0.05); C = probiotic fermented beverage produced from cow WPC and enriched with kiwi powder, G = probiotic fermented beverage produced from goat WPC and enriched with kiwi powder, S = probiotic fermented beverage produced from sheep WPC and enriched with kiwi powder, W = probiotic fermented beverage produced without WPC and enriched with kiwi powder.

**Table 2:** Pearson correlation matrix of the TPC and antioxidant capacities of probiotic whey beverages enriched with kiwi powder.

	TPC	DPPH	CUPRAC
TPC	1	0.820 **	0.395
DPPH	0.820 **	1	0.466 *
CUPRAC	0.395	0.466 *	1

** Means correlation is significant at 0.01 (*p* < 0.01). * Means correlation is significant at 0.05 (*p* < 0.05).

**Table 3:** Concentration of amino acids (mg/100 g) of probiotic whey beverages enriched with kiwi powder.

Item	Sample	Storage Days		
		1	14	28
Aspartic acid	C	ND	ND	ND
	G	6.30 ± 0.18 ^a^	6.17 ± 0.52 ^a^	5.42 ± 0.48 ^a^
	S	ND0.00 ± 0.00 ^Ba^	ND0.00 ± 0.00 ^Ba^	ND0.00 ± 0.00 ^Ba^
	W	ND0.00 ± 0.00 ^Ba^	ND0.00 ± 0.00 ^Ba^	ND0.00 ± 0.00 ^Ba^
Glutamic acid	C	141.00 ± 3.46 ^Aab^	144.89 ± 3.13 ^Aa^	129.46 ± 5.20 ^ABb^
	G	129.58 ± 4.25 ^Aa^	136.79 ± 2.76 ^Ba^	132.41 ± 1.79 ^ABa^
	S	126.72 ± 16.72 ^Aa^	135.96 ± 3.26 ^Ba^	126.69 ± 4.19 ^Ba^
	W	140.47 ± 1.32 ^Aa^	130.36 ± 1.72 ^Bb^	137.45 ± 1.66 ^Aa^
Asparagine	C	2.36 ± 0.11 ^A^	ND	ND
	G	2.49 ± 0.08 ^Aa^	2.99 ± 0.19 ^Aa^	2.39 ± 0.37 ^Aa^
	S	ND	1.61 ± 0.34 ^Ba^	1.16 ± 0.23 ^Bb^
	W	1.29 ± 0.09 ^Bb^	3.41 ± 0.20 ^Aa^	1.66 ± 0.13 ^Bb^
Serine	C	ND	ND	ND
	G	3.77 ± 0.25	ND	ND
	S	ND	ND	ND
	W	ND	ND	ND
Glutamine	C	ND	ND	ND
	G	2.28 ± 0.25 ^Ca^	1.72 ± 0.19 ^Ca^	2.22 ± 0.30 ^Ba^
	S	2.68 ± 0.06 ^Bb^	2.86 ± 0.17 ^Bb^	4.17 ± 0.04 ^Aa^
	W	3.64 ± 0.06 ^Ab^	6.78 ± 0.19 ^Aa^	3.78 ± 0.14 ^Ab^
Histidine	C	10.46 ± 0.19 ^Aa^	8.42 ± 0.52 ^Ab^	10.34 ± 0.66 ^Aa^
	G	7.98 ± 0.20 ^Bb^	8.83 ± 0.10 ^Aa^	7.89 ± 0.07 ^Bb^
	S	4.55 ± 0.16 ^Ca^	4.45 ± 0.29 ^Ba^	4.99 ± 0.14 ^Ca^
	W	3.18 ± 0.00 ^D^	ND	ND
Glycine	C	ND	ND	ND
	G	11.48 ± 0.43 ^b^	14.52 ± 0.28 ^a^	11.52 ± 0.49 ^b^
	S	ND	ND	ND
	W	ND	ND	ND
Threonine	C	9.98 ± 0.74 ^Bb^	7.95 ± 1.14 ^Bb^	15.31 ± 0.93 ^Ba^
	G	19.33 ± 0.61 ^Ab^	30.65 ± 0.62 ^Aa^	20.83 ± 0.25 ^Ab^
	S	ND	ND	ND
	W	ND	ND	ND
Arginine	C	ND	ND	ND
	G	ND	ND	ND
	S	70.66 ± 1.58 ^a^	57.79 ± 1.22 ^b^	52.16 ± 0.25 ^c^
	W	ND	ND	ND
Alanine	C	8.97 ± 0.25 ^Ba^	6.69 ± 0.02 ^Bb^	8.54 ± 0.01 ^Ba^
	G	11.95 ± 0.53 ^Ab^	14.89 ± 1.05 ^Aa^	13.96 ± 0.51 ^Aab^
	S	3.55 ± 0.42 ^Ca^	2.55 ± 0.45 ^Da^	3.26 ± 0.59 ^Ca^
	W	3.63 ± 0.25 ^Cab^	4.17 ± 0.16 ^Ca^	3.25 ± 0.28 ^Cb^
Tyrosine	C	16.24 ± 0.16 ^Bb^	14.55 ± 0.33 ^Bb^	20.92 ± 0.98 ^Aa^
	G	18.23 ± 0.59 ^Ab^	21.28 ± 0.88 ^Aa^	11.94 ± 0.77 ^Cc^
	S	14.95 ± 0.74 ^Ba^	12.66 ± 0.15 ^Cb^	10.46 ± 0.49 ^Cc^
	W	10.04 ± 0.16 ^Cc^	11.37 ± 0.40 ^Cb^	14.54 ± 0.33 ^Ba^
Cystine	C	6.32 ± 0.12 ^Ba^	4.56 ± 0.18 ^Bb^	5.79 ± 0.20 ^Ba^
	G	10.03 ± 0.84 ^Aa^	7.56 ± 0.31 ^Ab^	7.23 ± 0.16 ^Ab^
	S	4.01 ± 0.16 ^Ca^	4.36 ± 0.42 ^Ba^	3.66 ± 0.13 ^Ca^
	W	2.71 ± 0.07 ^Db^	3.96 ± 0.14 ^Ba^	3.67 ± 0.12 ^Ca^
Valine	C	31.00 ± 0.80 ^Ba^	19.40 ± 1.22 ^Bb^	20.24 ± 0.98 ^Cb^
	G	43.53 ± 1.87 ^Ab^	40.85 ± 0.02 ^Ab^	52.64 ± 0.72 ^Aa^
	S	20.69 ± 0.61 ^Cb^	20.01 ± 0.57 ^Bb^	28.79 ± 0.16 ^Ba^
	W	9.73 ± 0.13 ^Dc^	11.08 ± 0.64 ^Cb^	13.05 ± 0.13 ^Da^
Methionine	C	17.47 ± 0.25 ^Aa^	7.08 ± 0.07 ^Ab^	6.64 ± 0.21 ^Ab^
	G	10.86 ± 0.32 ^Ba^	6.30 ± 0.11 ^Bb^	6.01 ± 0.08 ^Bb^
	S	ND	ND	ND
	W	ND	ND	ND
Norvaline	C	ND	ND	3.06 ± 0.22 ^C^
	G	5.26 ± 0.61 ^b^	9.37 ± 0.41 ^Aa^	5.48 ± 0.10 ^Bb^
	S	ND	2.05 ± 0.16 ^Bb^	4.94 ± 0.11 ^Ba^
	W	ND	2.45 ± 0.18 ^Bb^	9.55 ± 0.42 ^Aa^
Tryptophan	C	5.41 ± 0.12 ^Aa^	2.28 ± 0.37 ^Bb^	1.73 ± 0.21 ^Cb^
	G	4.05 ± 0.25 ^Ba^	4.08 ± 0.25 ^Aa^	3.10 ± 0.06 ^Bb^
	S	2.49 ± 0.06 ^Ca^	1.58 ± 0.29 ^Bb^	2.66 ± 0.03 ^Ba^
	W	1.38 ± 0.15 ^Db^	1.78 ± 0.13 ^Bb^	3.94 ± 0.25 ^Aa^
Phenylalanine	C	16.48 ± 0.30 ^Aa^	7.51 ± 0.38 ^Bb^	6.75 ± 0.19 ^Cb^
	G	14.05 ± 0.71 ^Bc^	27.35 ± 0.38 ^Aa^	17.10 ± 0.32 ^Ab^
	S	ND	5.35 ± 0.00 ^Ca^	4.72 ± 0.00 ^Da^
	W	ND	4.41 ± 0.00 ^Db^	8.19 ± 0.00 ^Ba^
Isoleucine	C	21.38 ± 0.37 ^Ba^	3.90 ± 0.33 ^Dc^	5.63 ± 0.19 ^Db^
	G	26.78 ± 0.49 ^Aa^	18.34 ± 0.07 ^Ac^	20.55 ± 0.61 ^Ab^
	S	11.37 ± 0.12 ^Ca^	11.58 ± 0.42 ^Ba^	12.02 ± 0.18 ^Ba^
	W	3.77 ± 0.15 ^Dc^	5.02 ± 0.23 ^Cb^	8.95 ± 0.27 ^Ca^
Leucine	C	ND	16.11 ± 0.74 ^Ab^	18.62 ± 0.05 ^Aa^
	G	ND	ND	ND
	S	ND	ND	ND
	W	5.50 ± 0.40 ^b^	8.46 ± 0.48 ^Ba^	2.51 ± 0.24 ^Bc^
Lysine	C	18.25 ± 0.13 ^Aa^	9.32 ± 0.62 ^Ab^	6.51 ± 0.46 ^Bc^
	G	6.77 ± 0.56 ^Cb^	9.79 ± 0.35 ^Aa^	6.48 ± 0.42 ^Bb^
	S	9.10 ± 0.03 ^Ba^	6.91 ± 0.06 ^Bb^	5.22 ± 0.01 ^Bc^
	W	4.66 ± 0.30 ^Db^	5.54 ± 0.47 ^Cb^	9.25 ± 0.85 ^Aa^
Hydroxyproline	C	58.16 ± 1.03 ^Bb^	68.28 ± 1.20 ^Ba^	57.92 ± 0.45 ^Ab^
	G	14.42 ± 0.48 ^Dc^	86.68 ± 0.51 ^Aa^	28.56 ± 0.56 ^Db^
	S	179.15 ± 3.69 ^Aa^	37.41 ± 1.40 ^Cb^	43.32 ± 0.28 ^Bb^
	W	24.69 ± 2.33 ^Cb^	27.96 ± 0.28 ^Db^	37.76 ± 0.91 ^Ca^
Sarcosine	C	261.38 ± 1.41 ^Aa^	241.11 ± 2.18 ^Bb^	232.44 ± 0.87 ^Cc^
	G	256.43 ± 1.51 ^Bb^	246.53 ± 2.94 ^Ac^	263.83 ± 0.25 ^Ba^
	S	262.26 ± 0.17 ^Aa^	223.41 ± 0.19 ^Cc^	256.34 ± 1.63 ^Bb^
	W	232.30 ± 1.07 ^Cb^	211.20 ± 0.93 ^Dc^	284.22 ± 8.31 ^Aa^
Proline	C	72.42 ± 1.84 ^Bb^	69.01 ± 0.91 ^Db^	77.12 ± 0.90 ^Da^
	G	83.43 ± 1.40 ^Ac^	98.76 ± 1.56 ^Aa^	90.12 ± 0.44 ^Ab^
	S	87.13 ± 0.01 ^Aa^	88.30 ± 0.25 ^Ca^	87.68 ± 0.78 ^Ba^
	W	71.07 ± 1.93 ^Bc^	93.14 ± 0.98 ^Ba^	82.06 ± 0.40 ^Cb^
BCAAs	C	52.38 ± 1.17 ^Ba^	39.40 ± 1.63 ^Bc^	44.48 ± 0.74 ^Bb^
	G	70.31 ± 2.36 ^Ab^	59.19 ± 0.05 ^Ab^	73.19 ± 0.11 ^Aa^
	S	32.06 ± 0.73 ^Cb^	32.58 ± 0.42 ^Cb^	40.81 ± 0.02 ^Ca^
	W	18.99 ± 0.68 ^Db^	24.56 ± 0.08 ^Da^	24.51 ± 0.38 ^Da^
Total EAAs	C	130.40 ± 2.14 ^Aa^	81.95 ± 1.48 ^Bc^	91.74 ± 1.27 ^Bb^
	G	133.33 ± 0.85 ^Ab^	146.19 ± 0.28 ^Aa^	134.59 ± 0.05 ^Ab^
	S	48.19 ± 0.54 ^Bb^	49.86 ± 1.51 ^Cb^	58.40 ± 0.08 ^Ca^
	W	28.50 ± 1.13 ^Cc^	36.29 ± 0.52 ^Bb^	45.89 ± 1.48 ^Da^
Total FAAs	C	697.23 ± 3.37 ^Ba^	631.01 ± 0.37 ^Bb^	626.96 ± 8.73 ^Cb^
	G	688.94 ± 5.85 ^Bc^	793.42 ± 2.39 ^Aa^	709.65 ± 5.34 ^Ab^
	S	799.29 ± 11.72 ^Aa^	618.79 ± 0.64 ^Cc^	652.21 ± 3.27 ^Bb^
	W	518.02 ± 3.65 ^Cb^	531.08 ± 0.64 ^Db^	623.80 ± 8.57 ^Ca^

^a–c^ Means ± standard deviations in the same row with different superscript lowercase letters are significantly different (*p* < 0.05); ^A–D^ Means ± standard deviations in the same column with different superscript uppercase letters are significantly different (*p* < 0.05); C = probiotic fermented beverage produced from cow WPC and enriched with kiwi powder, G = probiotic fermented beverage produced from goat WPC and enriched with kiwi powder, S = probiotic fermented beverage produced from sheep WPC and enriched with kiwi powder, W = probiotic fermented beverage produced without WPC and enriched with kiwi powder. BCAAs = branched-chain amino acids, EAAs = essential amino acids, FAAs = free amino acids; ND = not detected.

**Table 4:** Pearson correlation matrix of the EAAs, FAAs, TPC, and antioxidant capacities of probiotic whey beverages enriched with kiwi powder.

	EAAs	FAAs	TPC	DPPH	CUPRAC
EAAs	1	0.615 **	0.821 **	0.846 **	0.221
FAAs	0.615 **	1	0.416 *	0.572 **	0.323
TPC	0.821 **	0.416 *	1	0.820 **	0.395
DPPH	0.846 **	0.572 **	0.820 **	1	0.466 *
CUPRAC	0.221	0.323	0.395	0.466 *	1

** Means correlation is significant at 0.01 (*p* < 0.01). * Means correlation is significant at 0.05 (*p* < 0.05).

## Data Availability

The datasets generated for this study are available on request to the corresponding author.
